# Supplementary material for: Polymeric Composites with Silver (I) Cyanoximates Inhibit Biofilm Formation of Gram-Positive and Gram-Negative Bacteria
Source: Polymers (Basel). 2019 Jun 9;11(6):1018. doi: 10.3390/polym11061018 (PMC6631325; doi:10.3390/polym11061018)
Supplement: Supplementary file 1 [file polymers-11-01018-s001.pdf]

Pages of electronic supporting information, ESI, for the paper:

Polymeric composites with Silver (I) Cyanoximates Inhibit Biofilm Formation of Gram-positive and Gram-negative bacteria.

S.R. Lotlikar[1], E. Gallaway[1], T. Grant[1], S. Popis[2], M. Whited[2], M. Guragain[1], R. Rogers[1], S. Hamilton[1], N.G. Gerasimchuk[2]*#, and M.A. Patrauchan[1]*#

1 Department of Microbiology and Molecular Genetics, Oklahoma State University, Stillwater, OK, USA

2 Department of Chemistry, Missouri State University, Springfield, MO, USA

# - corresponding authors from *chemical* and *biological* sides of studies respectively

Table S1

Components of the light-curable acrylate polymer that is currently used in dental practice and was used in current studies: details.

1. Triethylene glycol dimethacrylate (TEGMA), contains 80-120 ppm MEHQ as inhibitor, 95%, CAS Number 109-16-0, Lot #STBD8159V.
2. Diurethane dimethacrylate (UDMA), mixture of isomers contains 225 ppm ±25 ppm topanol as inhibitor, ≥97%, CAS Number 72869-86-4, Lot #MKBK4814V.
3. Bisphenol A glycerolate dimethacrylate glycerol/phenol 1(Bis-GMA), CAS Number 1565-94-2, Lot #MKBV3327V.
4. Silicagel (SiO2) Hollow, non-porous glass beads, 9-13 μm particle size, CAS Number 65997-17-3, Lot # MKBL4299V.
5. Camphorquinone (CPQ) 97%, CAS #10373781, Lot #09003AQV.
6. Ethyl 4-(dimethylamino) benzoate (EDBMA), ≥99% also known as Parbenate CAS# 10287533, lot# MKBG9133V.

Table S2

Comparison of the composition of acrylate light curable composite currently used in dentistry and in our experiments in order to address desire for more flovability of mixtures.

Chemicals	Chemicals Structures	Original Composite % wt/wt	Our Composite % wt/wt
(TEGMA) Triethylene glycol dimethacrylate,<br><br>Contains 80-120 ppm MEHQ as inhibitor, 95%; CAS#109160 (Sigma-Aldrich).	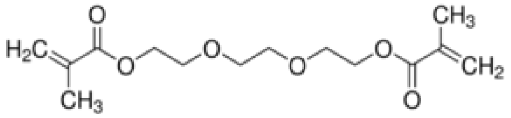	15%	**20%**
(UDMA) Diurethane dimethacrylate,<br><br>Mixture of isomers contains 225 ppm ±25 ppm topanol as inhibitor, ≥97%; CAS#72869864 (from Sigma-Aldrich).	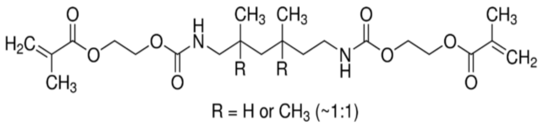	15%	20%
(Bis-GMA) Bisphenol A glycerolate dimethacrylate glycerol/phenol 1,<br><br>CAS#1565942 (from Sigma-Aldrich).	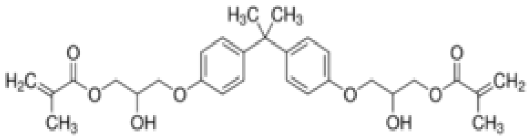	15%	15%
(SiO_2) Glass spheres, 9-13 µm size, hollow, spherical non-porous glass beads. CAS# 65997173 (from Sigma-Aldrich).	SiO_2	55%	**45%**

(CQ) Camphorquinone 97%,<br><br>CAS#10373781, Lot #09003AQV from Sigma-Aldrich.	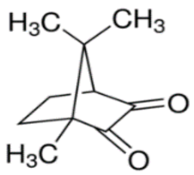	0.5%	0.5%
(EDBMA) Ethyl 4-(dimethylamino)benzoate ≥99% aka Parbenate<br><br>CAS#10287533, lot# MKBG9133V from Sigma-Aldrich.	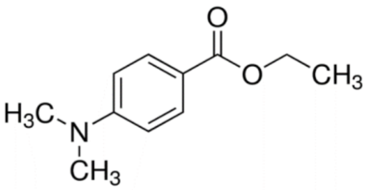	0.5%	0.5%

Changes in the “recipe” from the original dental composition and adopted in this experiment are indicated by the red color.

Figure S1

A – general view of the thermal analyzer showing blow torch for crucible cleaning and tools; **B** – cleaning alumina crucible with flame; **C** – opened furnace view showing two beams with alumina crucibles for differential analysis (darkened for sample, lighter – empty – for control).

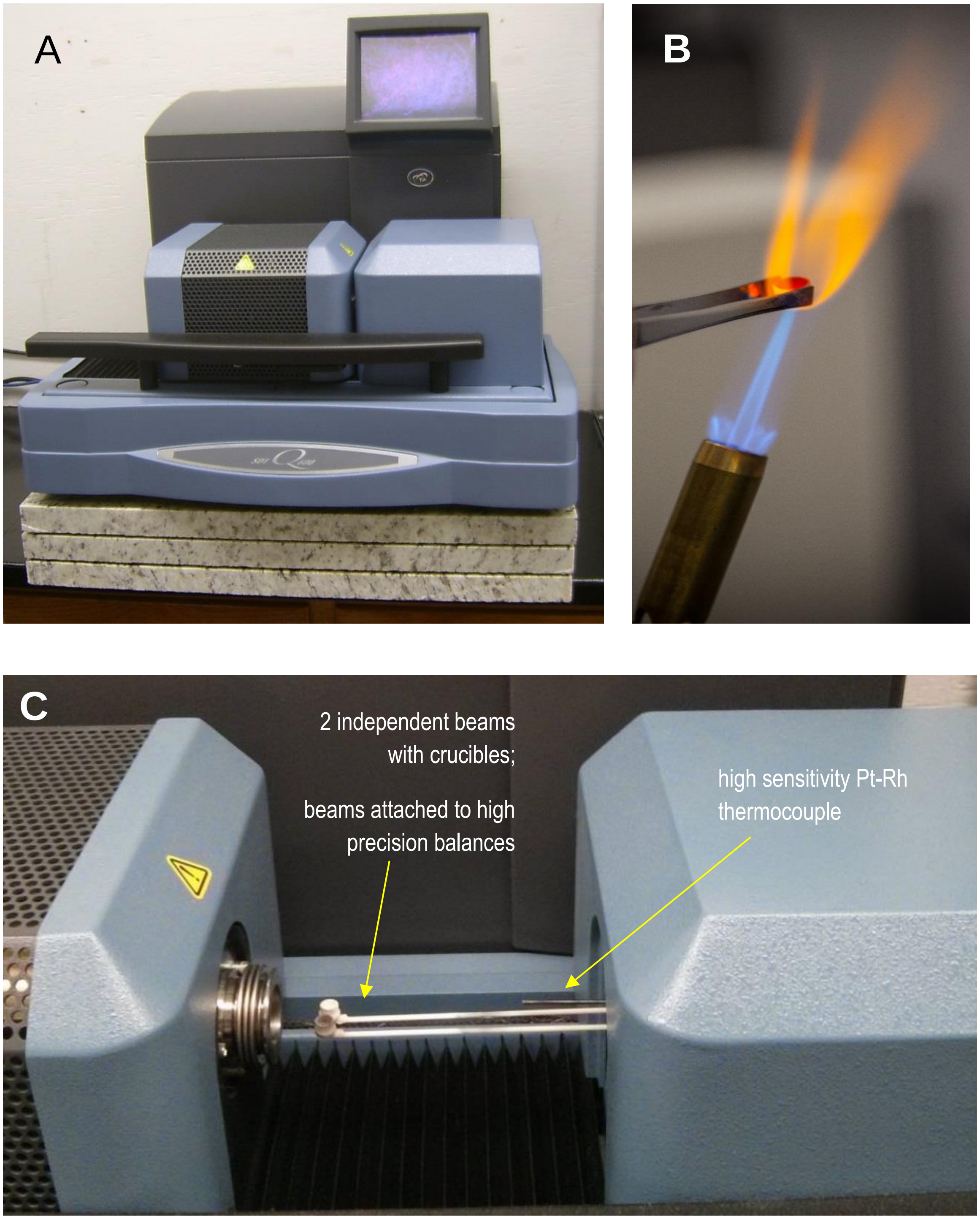

Figure S2

Full thermal analysis profile for a sample of Ag(2PCO).

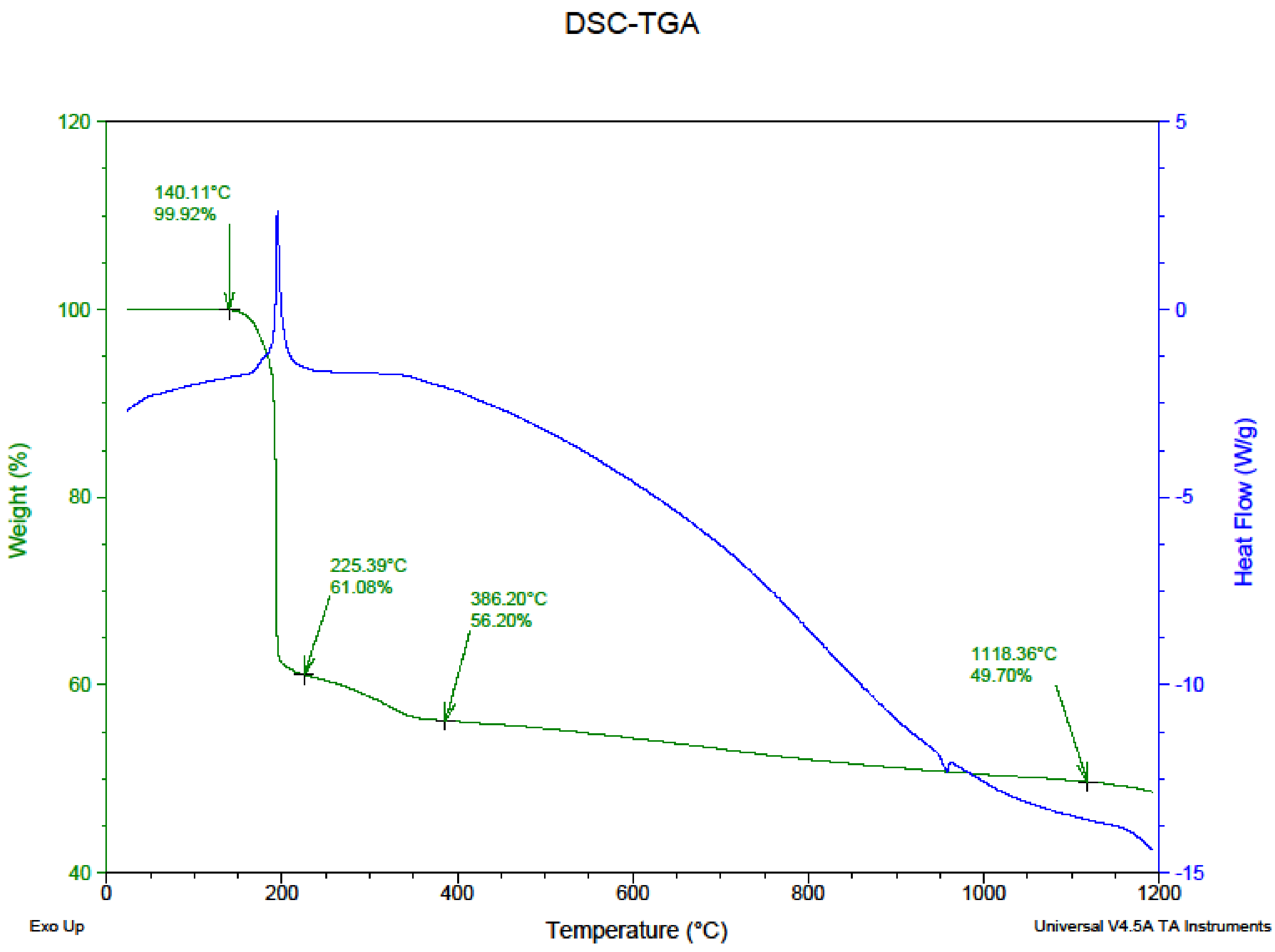

Figure S3

A fragment of the low temperature region of thermal analysis plot for a sample of Ag(BCO). Shown great range of thermal stability of the somplex well above necessary themperature for sterilization purposes.

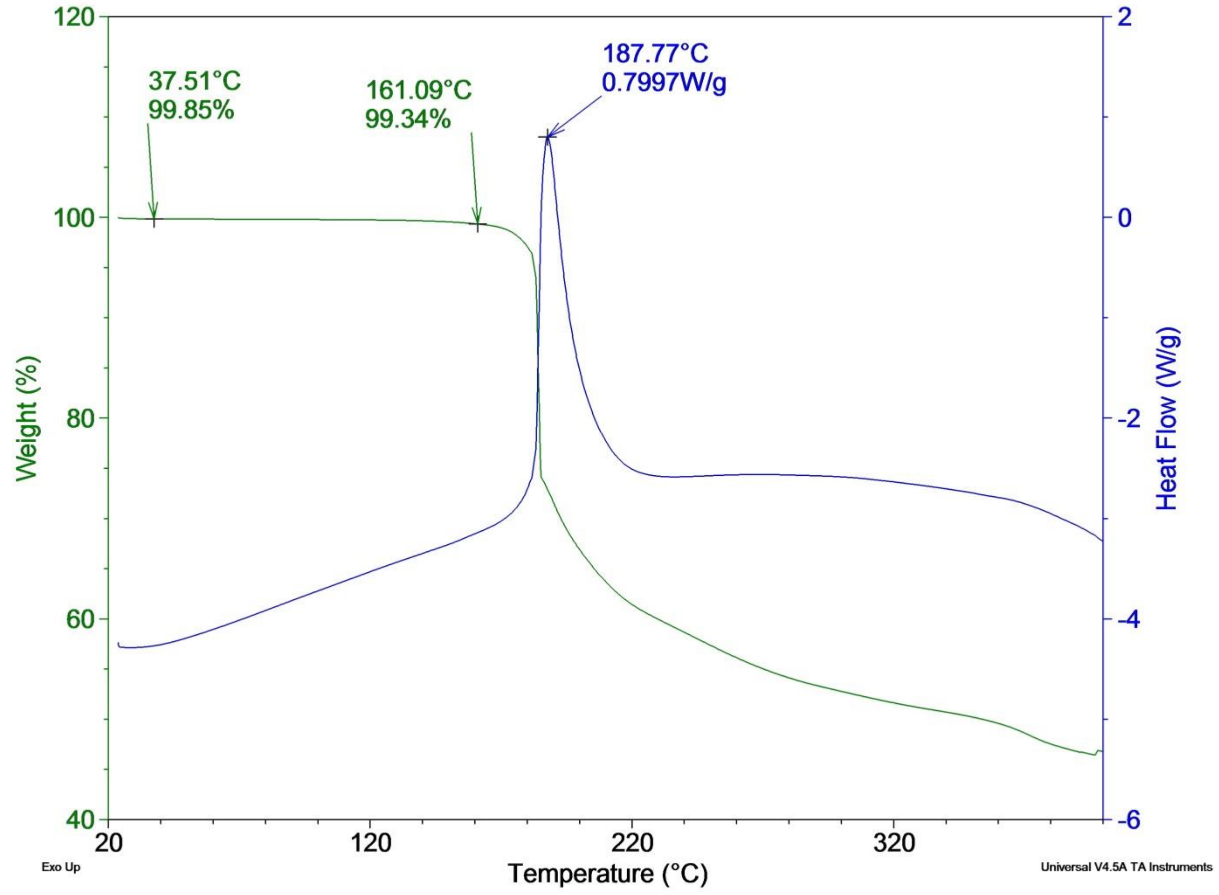

Ag(BCO)

Figure S4

A fragment of the high temperature region of thermogramm for a sample of Ag(ECO). Shown here melting of elemental silver in the alumina crucible. Metallic silver is the final product of thermal decomposition process of studied under nitrogen atmosphere AgL.

Ag(ECO), silver melting part

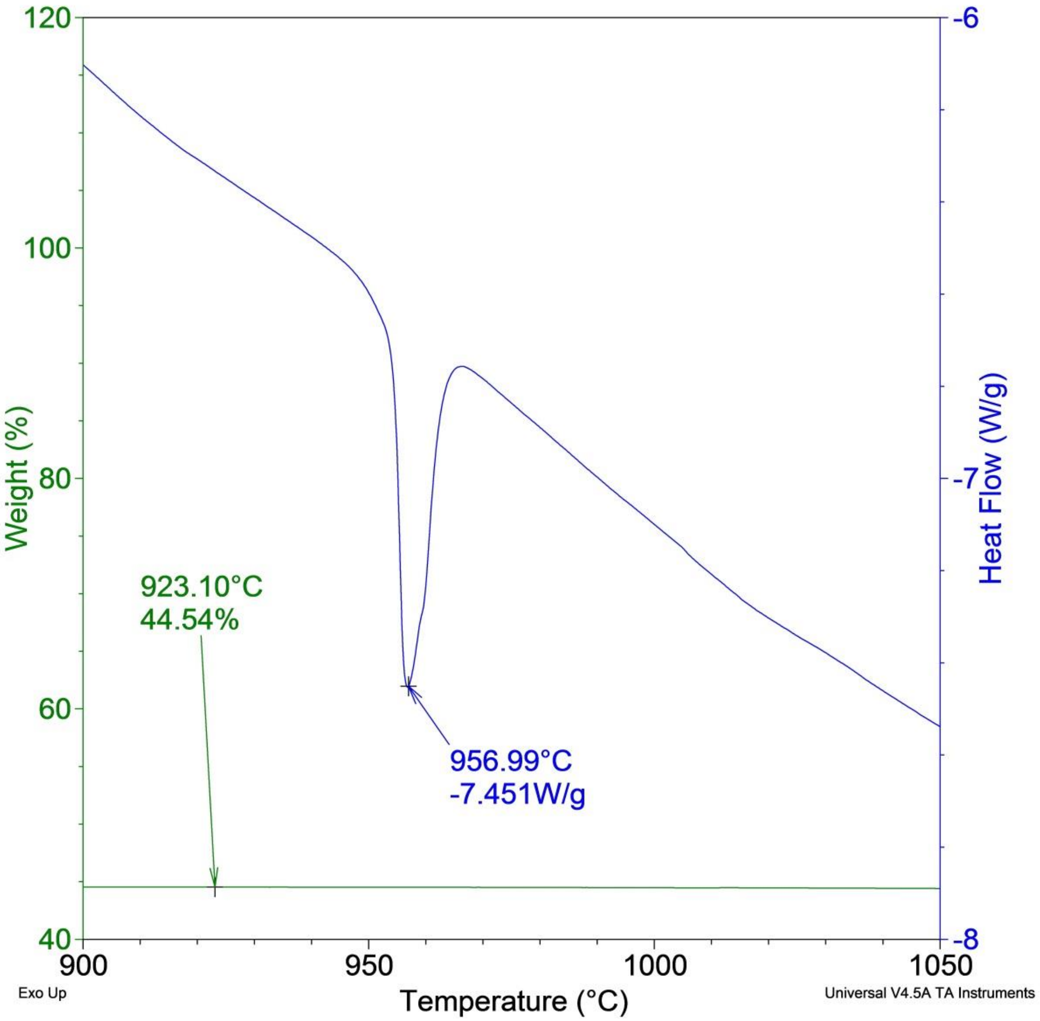

Figure S5

A – optical microscope used in assessment of samples homogienity and taking photographs at 40x;
B – actual photos of several silver(I) cyanoximates.

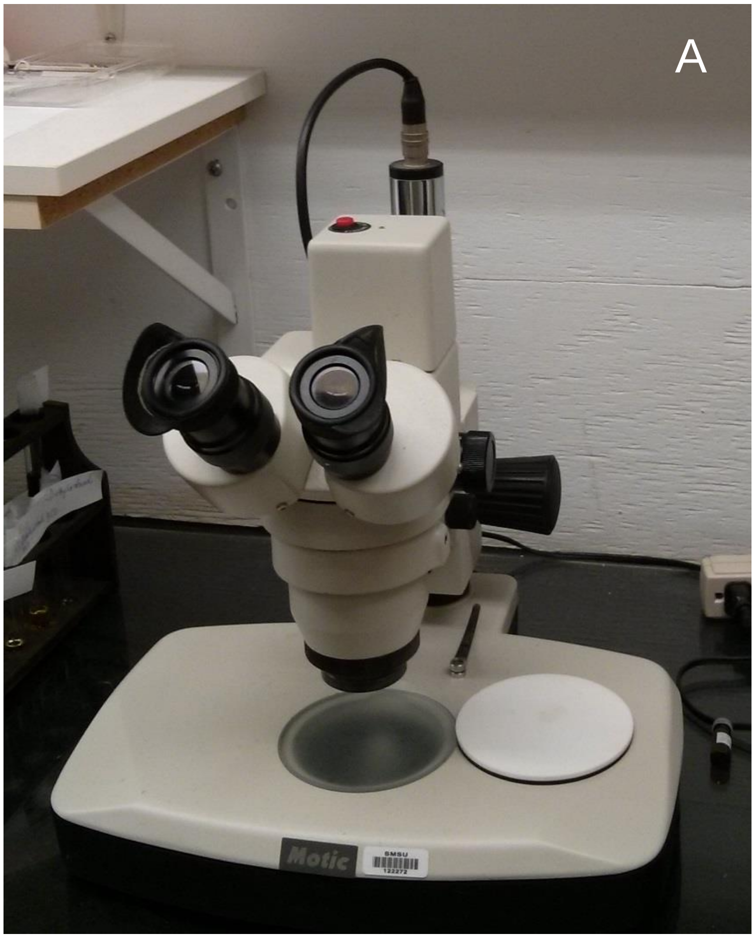

B

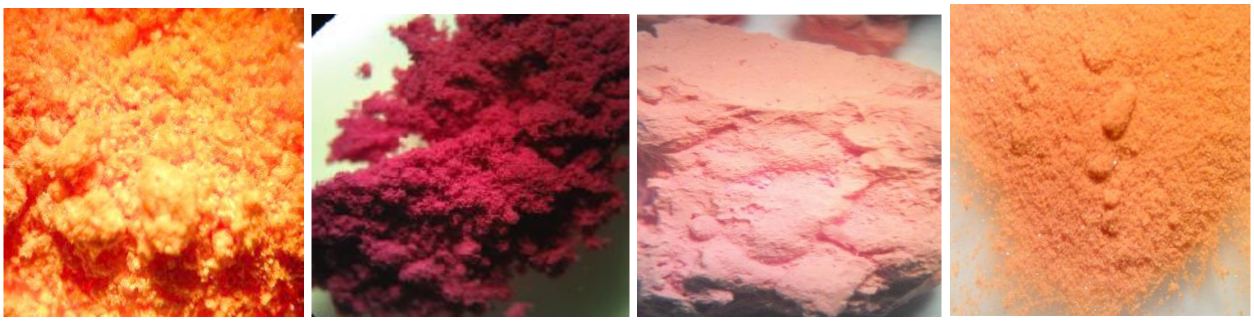

Ag(ECO) **Ag(BCO)** **Ag(PiCO)*** **Ag(PiCO)****

red polymorph yellow polymorph

Figure S6

_ Actual photographs of powdery samples of two polymorphs of silver(I) pivaloylcyanoximates.

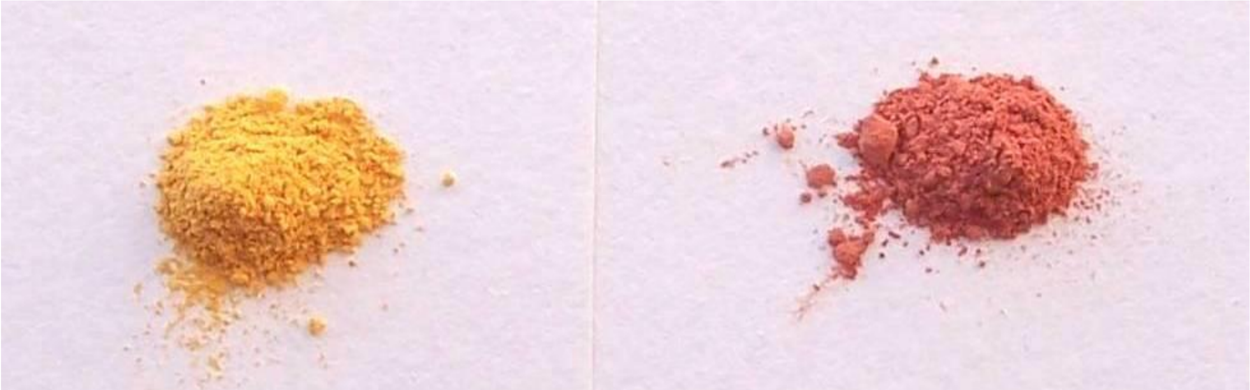

Ag(PiCO) yellow polymorph Ag(PiCO) red polymorph

Table S3. Properties of synthesized silver(I) cyanoximates.

Complex	Color	Yield, %	Available quantity, g
Ag(CCO)	canary-yellow	95	10.2
Ag(ACO)	yellow	98	5.0
Ag(ECO)	yellow-orange	95	5.1
Ag(BCO)	red-purple	95	3.1
Ag(PiCO) *	pink	95	5.2
Ag(BIHCO)	pale-yellow	93	2.5
Ag(DCO)	dark-yellow	82	1.1
Ag(BOCO)	orange-brown	96	4.0
Ag(BIMCO)	yellow-brown	94	3.2
Ag(BTCO)	light-brown	93	4.1
Ag(PiPCO)	lemon-yellow	89	0.96
Ag(MCO)	bright-yellow	83	1.24

*- red polymorph

Table S4. Data of elemental analyses for AgL complexes.

Complex	Elemental composition, %, calculated (found)		
	C	H	N
Ag(CCO)	17.86 (17.84)	0 (0)	20.71 (20.81)
Ag(ACO)	16.49 (16.38)	0.90 (0.92)	19.08 (19.11)
Ag(ECO)	24.10 (24.12)	1.90 (2.02)	11.07 (11.25)
Ag(BCO)	37.98 (38.47)	1.80 (1.79)	9.71 (9.97)
Ag(PiCO)·0.5 H_2O**	31.13 (31.05)	3.73 (3.35)	10.37 (10.29)
Ag(BIHCO)·0.5 H_2O	35.79 (35.93)	2.00 (1.94)	18.55 (18.42)
Ag(DCO)·H_2O	22.58 (22.88)	3.03 (2.91)	15.80 (15.91)
Ag(BOCO)	36.77 (37.40)	1.37 (1.60)	14.29 (14.01)
Ag(BIMCO)·H_2O	36.95 (36.71)	2.79 (2.56)	17.24 (17.14)
Ag(BTCO)	34.86 (34.85)	1.30 (1.51)	13.55 (13.50)
Ag(PiPCO)	33.36 (33.39)	3.50 (3.37)	14.59 (14.36)
Ag(MCO)	28.99 (28.92)	2.78 (2.63)	14.49 (14.25)

**- yellow polymorph

Figure S7

The integrating sphere for recording SDR of powdery AgL (**A**), white Nylon filters used as support for complexes' powders (**B**), and assembled spectrophotometer ready for studies (**C**).

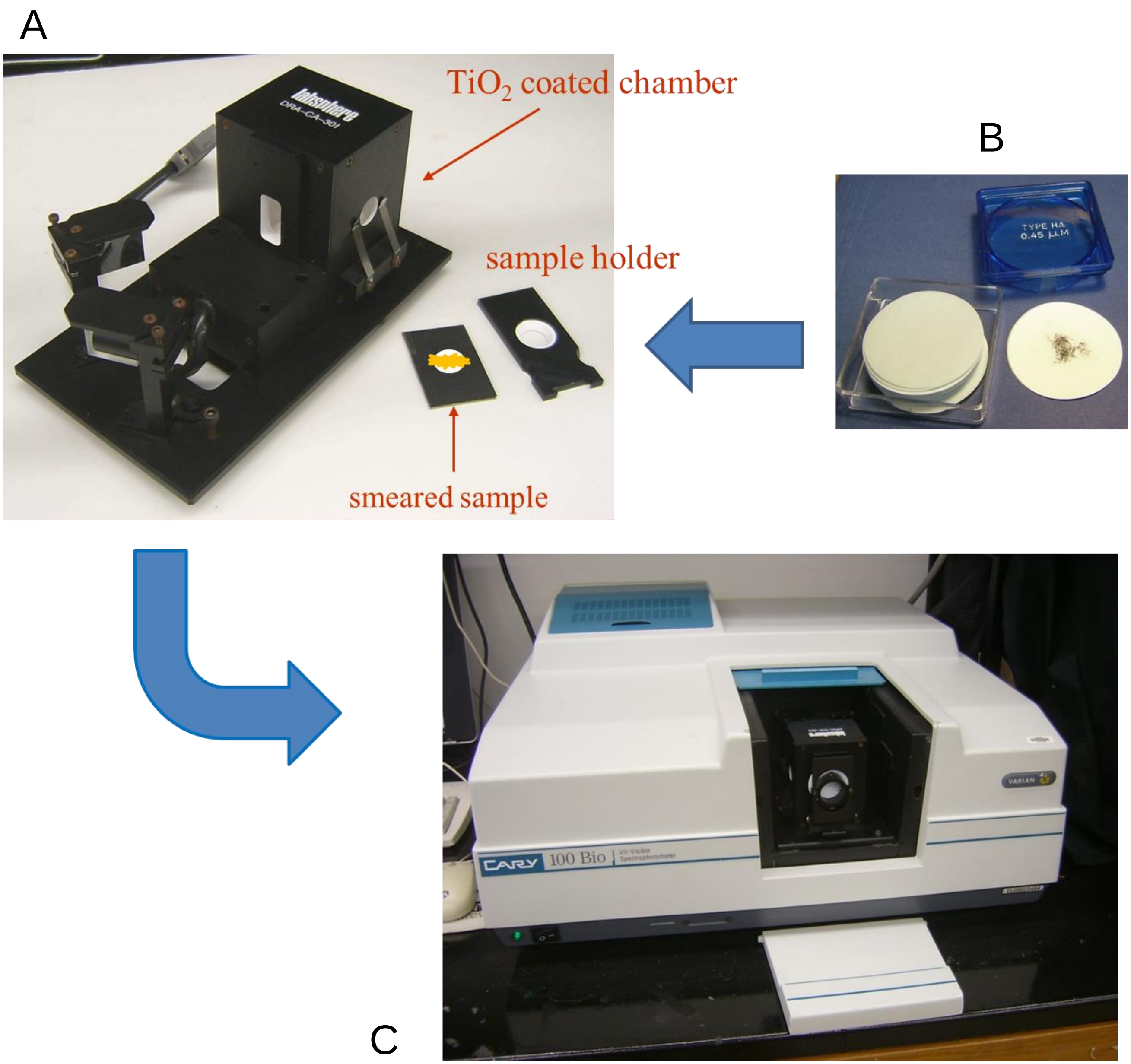

Figure S8

A – standard light sourse (400 nm, blue LED) used in dental practice to cure acrylate composites; **B –** solidified samples of the flowable composite mixed with Ag(CCO) and cured within 40 sec (left), and Ag(ACO) cured within 140 sec (fright).

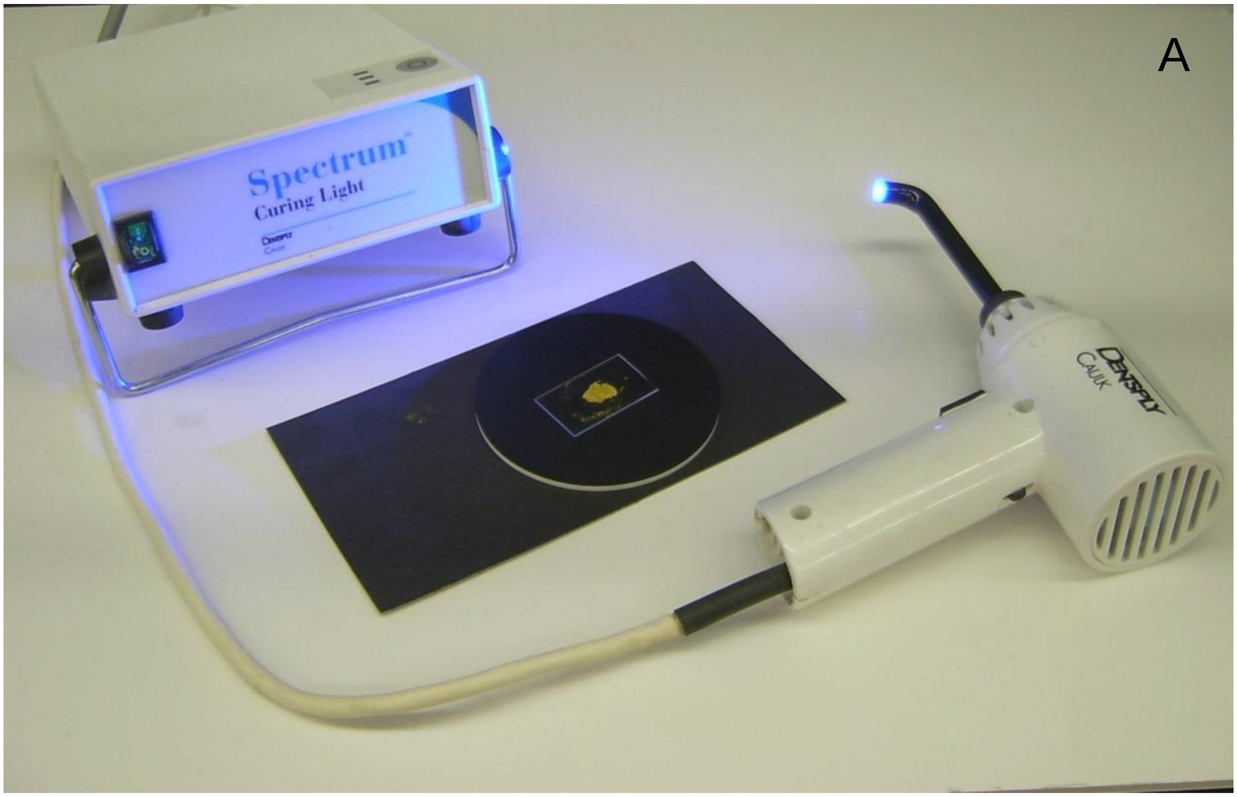

B

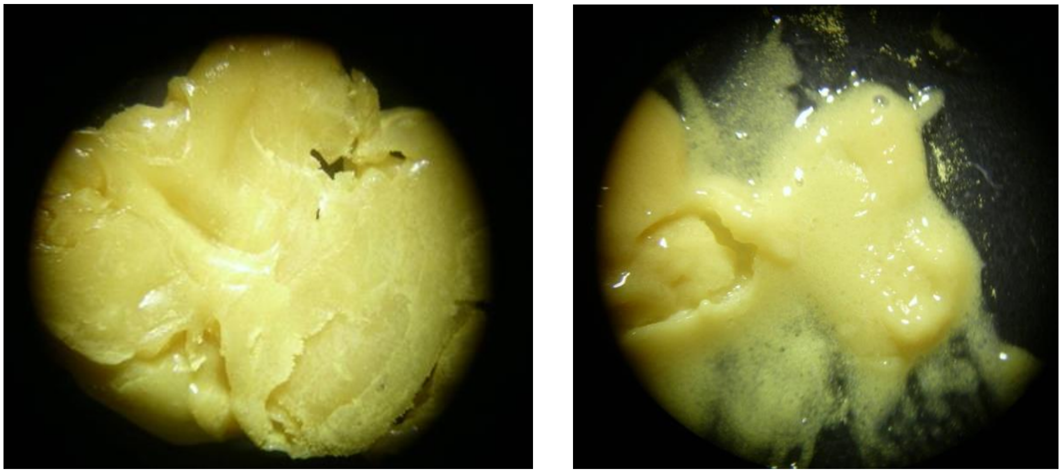

Figure S9

Filling wells of the 96-wells plate with polymeric composite containing silver(I) cyanoximates via syringe.

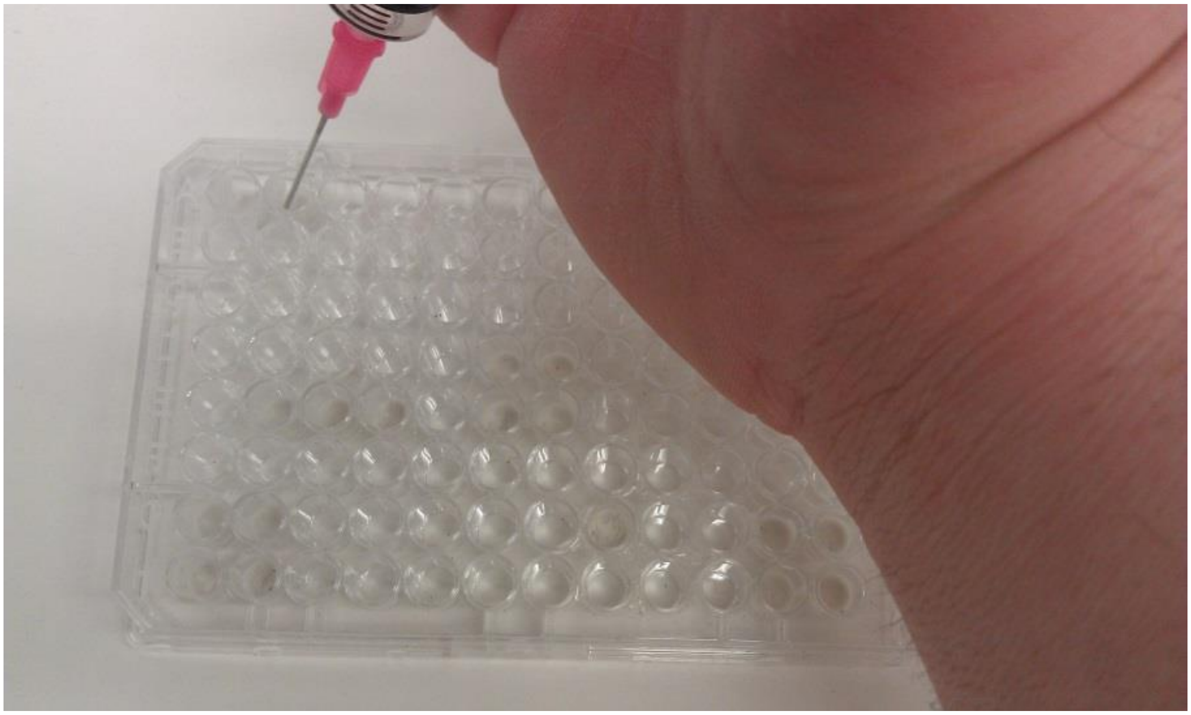

Figure S10

Actual appearance of prepared polymeric composite filled into 96-wells plate for microbiological testing prior to the light-induced polymerization.

Top view:

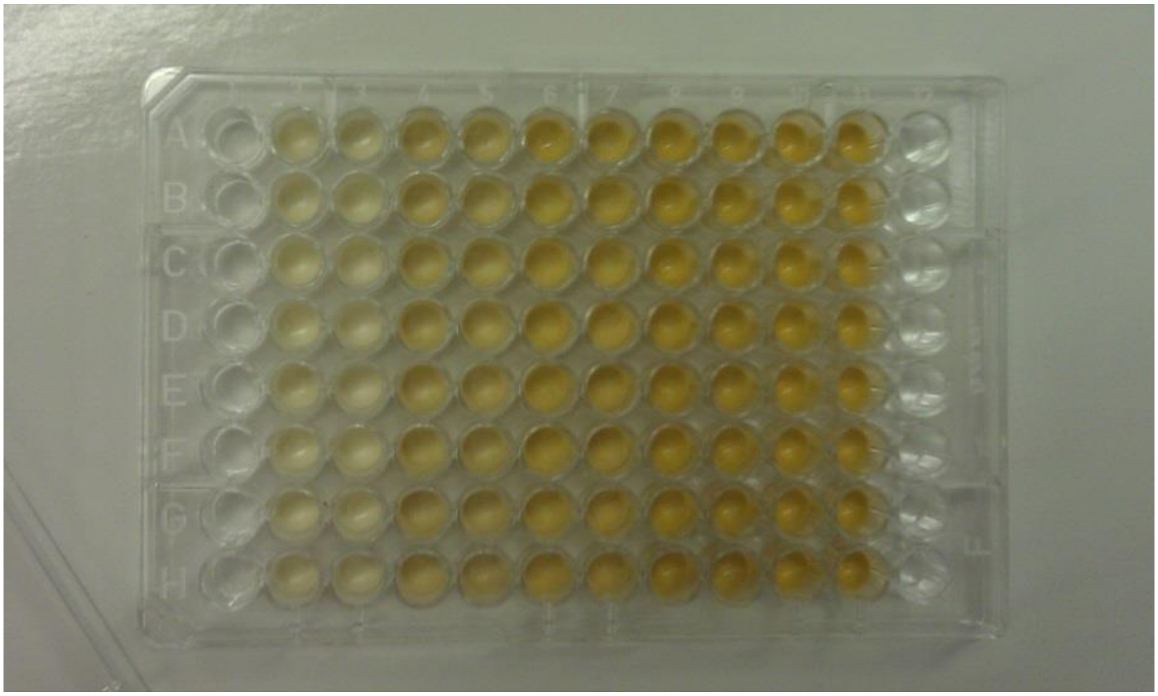

Side view:

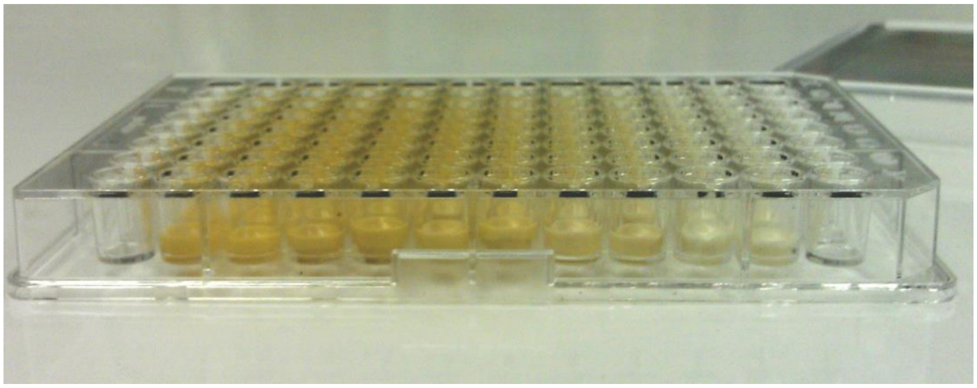

Figure S11

Experimental setup for the photopolymerization of composites containing *yellow colored* AgL using the high intensity halogen lamp.

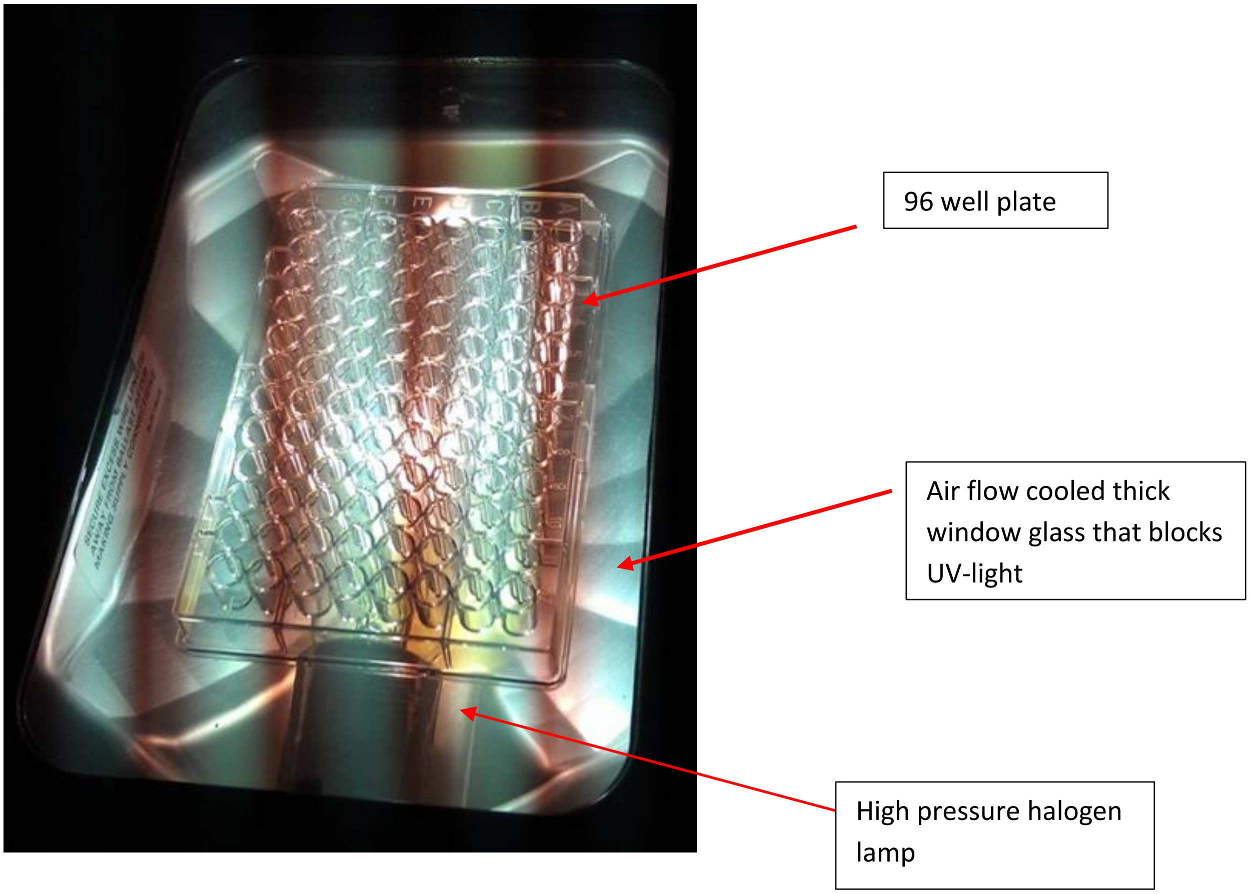

Figure S12

Silver(I) ions leaching experiments: design and important details.

Three Silver(I) complexes – AgCCO, Ag(ACO) and Ag(BTCO) at 5% w/w concentrations – were selected for making polymeric composites for the leaching experiments. Two reasons were behind those choices: A) these cyanoximes represent a range of hydrophilic and hydrophobic ligands used to bind metal cations; B) five percent by weigh is the higest load, highest concentration of the metal compounds that was used in antimicrobial studies. Thus, if a leaching of silver ions to be happeneing, then at this concentration it would be possible to accurately measure it. The non-halogenated buffers (to avoid forced leaching of Ag+ due to its high affinity to halogenide ions and formation of poorly soluble precipitates) were selected to be: TRIS, and MOPS.

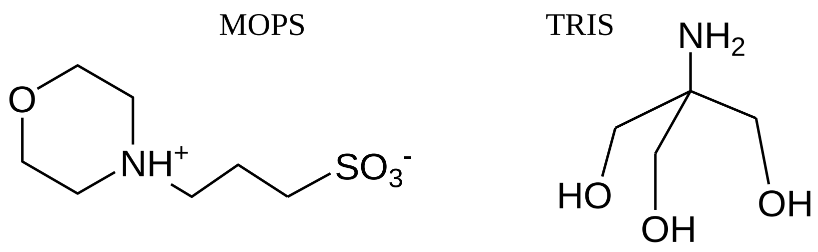

The whole experiment consisted of five parts:

1) Preparation of flowable polymeric acrylate composites with embedded at 5% w/w silver(I) cyanoximates;
2) Delivery of mixtures into the pre-made form for casting;
3) Curing mixtures with light until solid pellets are formed;
4) Exposure of pellets to non-halogenated buffer solutions at pH~7 and take aliquots within certain time intervals;
5) Analyses of colelcted aliquots using the analytical method ICP.

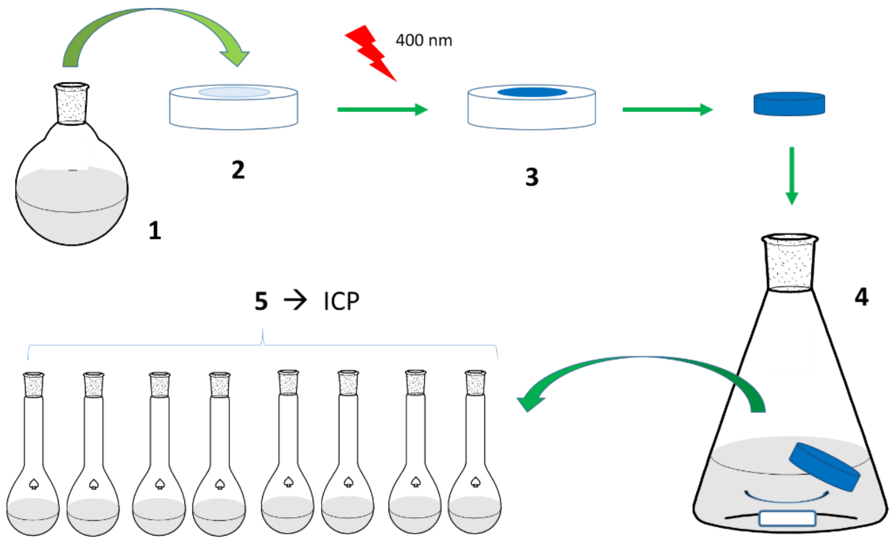

Table S5

Procedure of Making the Composite:

Abbreviations for used ingredients can be found in Table S3, S4 above.

Five percent by weight Silver(I)cyanoximates were mechanically mixed (embedded? extruded?) into our modified for better flowability and handling acrylate polymeric composite.

Thus, dental composite mixture was prepared as follows: 20% w/w of TEGMA, 20% w/w of UDMA, 15% w/w of Bis-GMA combined in a beaker, and mechanical mixed at 50-75 rpm for 10 minutes between additions. Then 45% mass by mass add SiO_2 added slowly in increments of ~1/8 from total mass to ensure the homogeneity. The mixing process introduces air bubbles into the mixture at this point, and a slower speed of the mechanical mixer was chosen to reduce the amount of air bubbles. Bulk samples loaded into syringes and stored under refrigeration. Photo activators, 0.5 % mass by mass CPQ and 0.5% mass by mass EDBMA, and 5% mass by mass silver complex with a small amount of SiO_2 combined and crushed into a fine powder in a motor and pestle to ensure a smaller crystal size, and homogeneity throughout the final pellet. In the dark, under mechanical mixing of 50-75 rpm, in beaker combine, refrigerated dental solution and finely powder photo activators and silver ligand for ten minutes. Loaded activated dental composite into syringes.

The mixture for deposition in forms for casting to make polymers with 5% Ag(CCO) contained 0.889 g of the complex, while for 5% $AgNO_3$ (used as control) it was 0.607 g of salt, and for 5% Ag(ACO) it was 0.6883 g and for 5% Ag(BTCO) - 0.6645 g of complexes respectively.

Polymeric Composites Casting into pellets:

A cylinder shape forms for a pellets was made from a cleat plastic tubing (PVC) by cutting approximately one centimeter of length with a 1 cm in diameter opening. These forms were attached to a piece of cardboard by a superglue. Thus, a cast was made. The pellet is made by liquid dental composite injected into the cast by syringe in layers of approximately 1-2 mm thick. Each layer was cured by dental lamp for one minute increments, for a total of 10-12 minutes per layer until polymerized into a solid, before next layer is applied.

The “solidness” of the obtained composite was checked by the needle probe used in medical dental practice.

Total five pellets (including one without any AgL embedded – for control!) were produced for each of the studied Ag-cyanoximate: Ag(CCO), Ag(ACO) and Ag(BTCO).

Table S6

Leaching Experiments:

Prepared as above solid dental composites in the sahepe of cylindrical casted pellets were placed in different buffered systems (MOPS or TRIS) and their pH was adjusted to ~7.0. Conventional orbital shaker used frequently in biological experiments was employed to agitate the mixture. Experiments conducted at temperatures between 26°C to 30°C. Each pellet was placed into the 500 mL Erlenmeyer flask that contained 100 mL of buffer solution, and then shaken in the dark on an orbital shaker in constant motion. Thus, an orbital shaker, preset at continuous mode of 100 rpm during entire experiment was positioned in a dark box to avoid possible photoreduction of leached silver(I) ions to metalic form which could skew the measurements. The total length of the leaching experiment was 168 hours.

A newly calibrated automatic pipette of 1000 µL capacity was used to transfer aliquots. These were of 1 mL and 2 mL in volume, and were placed into polyethylene tubes with screw caps, diluted with 10 mL of acidified DI water, and placed in the dark waiting to be analyzed on silver content.

Silver(I) ions content measurements:

As stated above, aliquots that were taken at predetermined time intervals, were then analyzed using an ICP method on an instrument which has been calibrated and programed for a set of experiments at one period per trial (for different buffers).

Calibrated with ICP standards of silver(I) ions at 50.00 ppm, 10.00 ppm, 5.00 ppm, 1.00 ppm, 0.100 ppm concentrations prepared according to standard laboratory procedure. All these standards were made with commercially available Silver ICP Standard. Dilution was done with acidified double deionized water (acidified DDI-water) made by adding a solution of 1 M nitric acid into 1 L double deionized water (DDI-water) to pH = 5.5.

Typical programed conditions for the experiment are the following: 240 kPa pressure, stabilization time 5 seconds, 20 second sample delay, 1 second run/integration, 5 replicates averaged, at filter position 6, order 1, power 1.00 kW, plasma 15.0 L/*min, Aux 1.50 (L/min), with a pump speed of 15.0 rpm. In the second set of experiments (March) changed 1 second run/integration, to 3 second run/integration to increase clarity of raw data, increased time for averaging, therefore increase accuracy of results. Provided a final correlation consistent (CC) limit of 0.9999.

Figure S13

General trends in changes of vibrations in the C-N-O fragment in cyanoximes / nitroso-compounds.

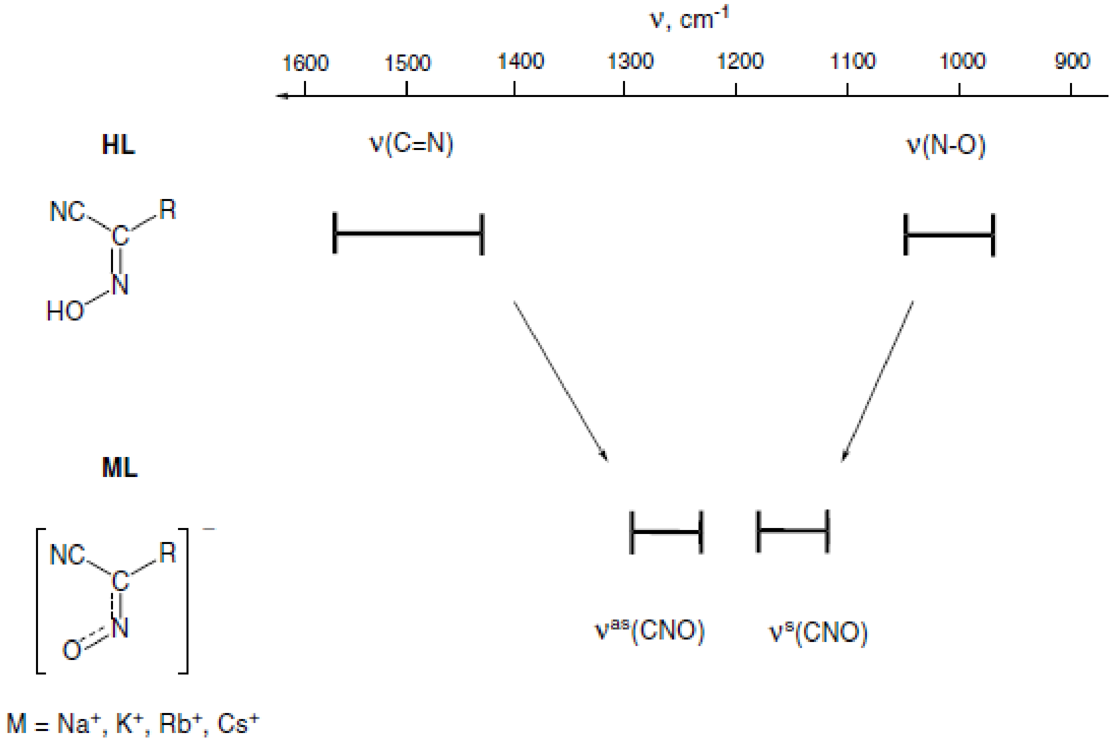

Gerasimchuk, N.; Goeden, L.; Durham, P.; Barnes, C.; Cannon, J.F. Synthesis and Characterization of the First Disubstituted Arylcyanoximes and their Several Metal Complexes." *Inorg. Chim. Acta*, (2008), *361*, 1983-2001.

Figure S14

Solid state electronic spectrum of Ag(2PCO).

Diffusion reflectance spectrum of pure powder of the complex (blue trace), and transformed into more convenient absorbance mode (red trace). Arrows indicated *n* → p* transitions in the nitroso- chromophore.

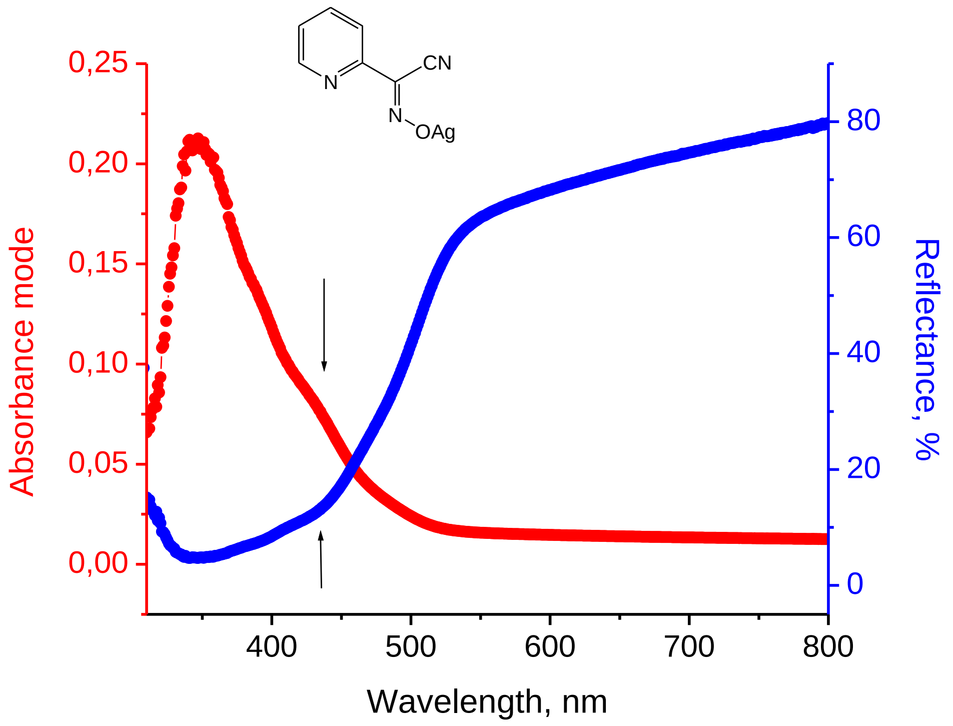

Figure S15

Solid state electronic spectrum of Ag(BCO).

Diffusion reflectance spectrum of pure powder of the complex (blue trace), and transformed into more convenient absorbance mode (red trace). Arrows indicated *n* → p* transitions in the nitroso- chromophore.

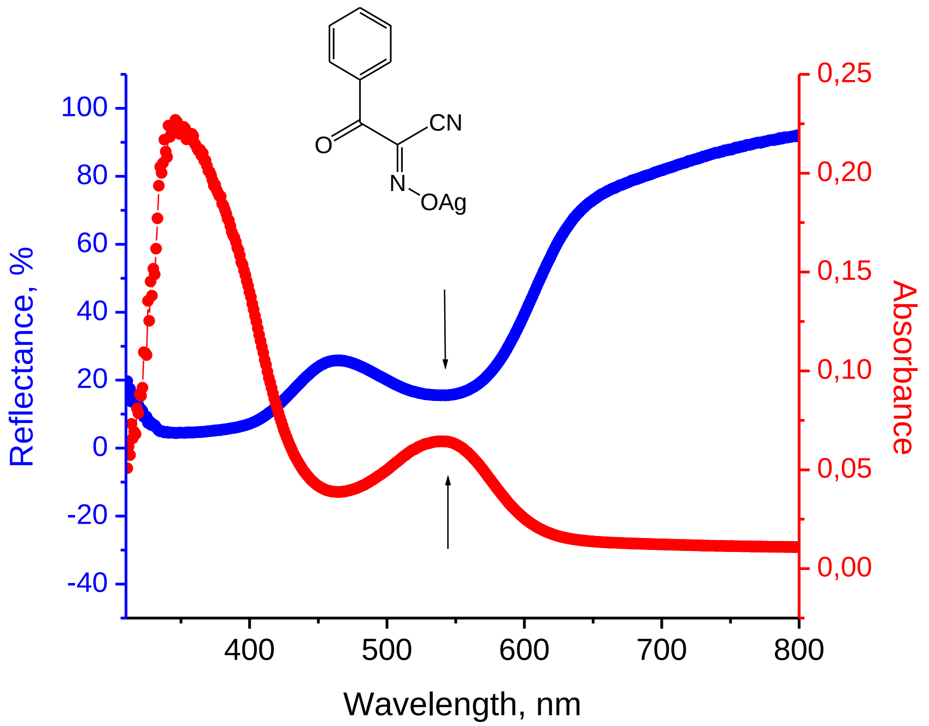

Figure S16

Solid state electronic spectrum of Ag(ECO).

Diffusion reflectance spectrum of pure powder of the complex (blue trace), and transformed into more convenient absorbance mode (red trace). Arrows indicated *n* → p* transitions in the nitroso- chromophore.

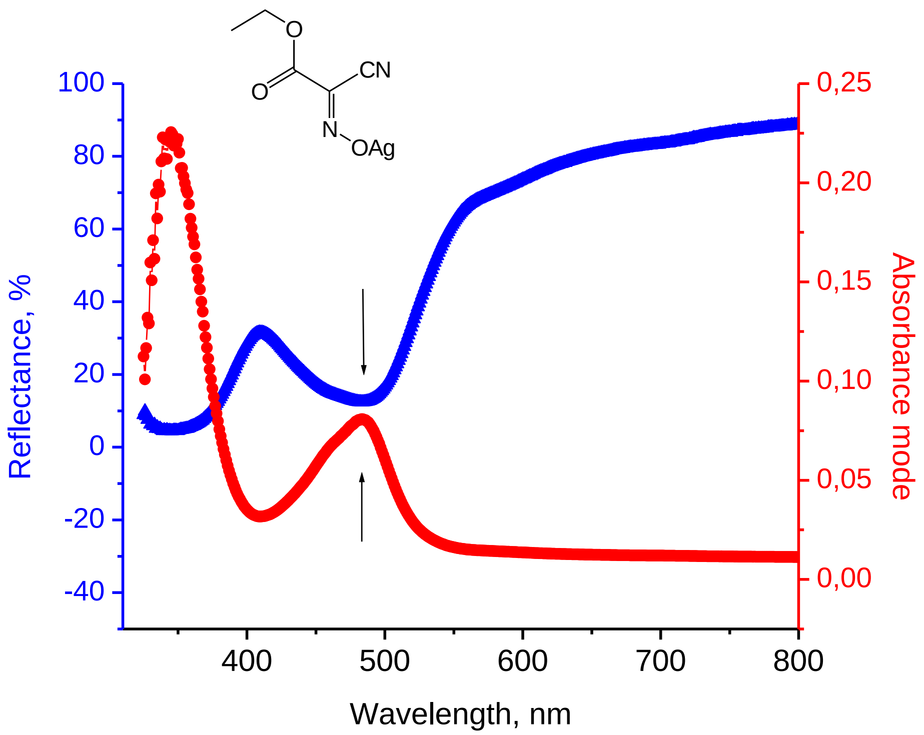

Figure S17

Results of a Silver(I) ions leaching experiment carried out in water at room temperature and no buffers added. Solid support consisted of only acrylate polymeric composite that was used as control. Other solid supports contained Silver nitrate, as the most commonly used silver antimicrobial compound, and two Silver(I) cyanoximates of different hydrophobicity/ hydrophylicity.

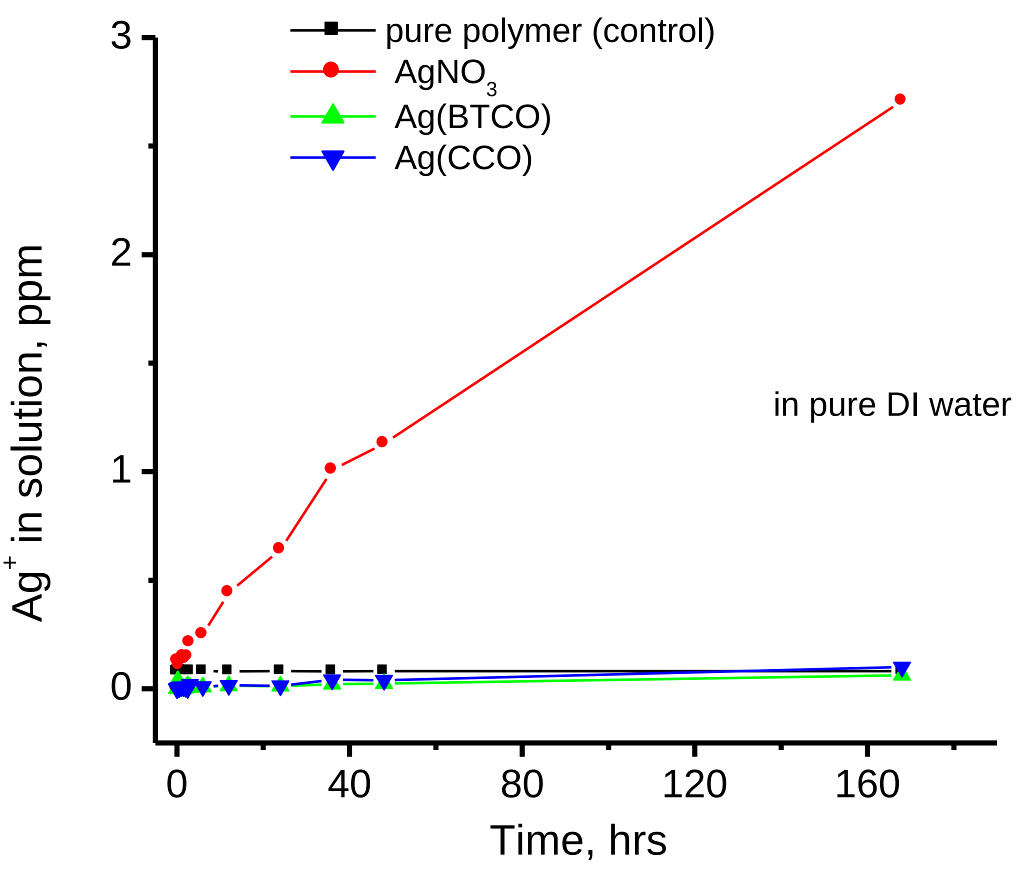

Figure S18

Results of thermal analyses studies of solid polymeric composite without embedded Ag-cyanoximate **(A)**, and with 5% w/w of the Ag(ACO) **(B)**. Red line shows the end of safe T zone.

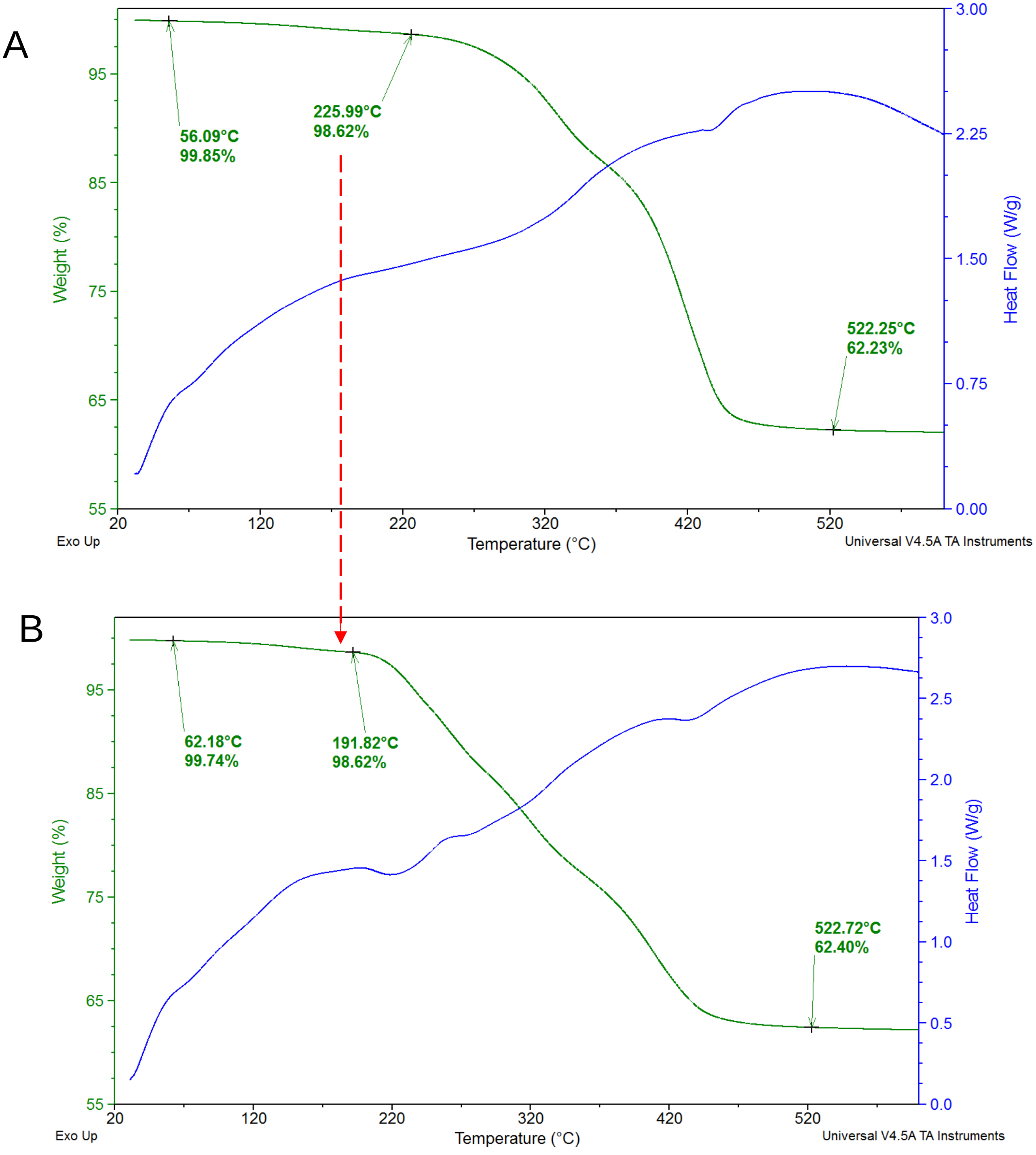

Figure S19

Apparatus - Instron 3369 universal testing machine at a strain rate of 2.5 mm/mim, using a 50 kN load cell following a testing procedures in the spirit of ASTM D1621−04a (Standard Test Method for Compressive Properties of Rigid Cellular Plastics) – used for measurements of mechanical strength of samples of polymeric composite with embedded Ag-complexes.

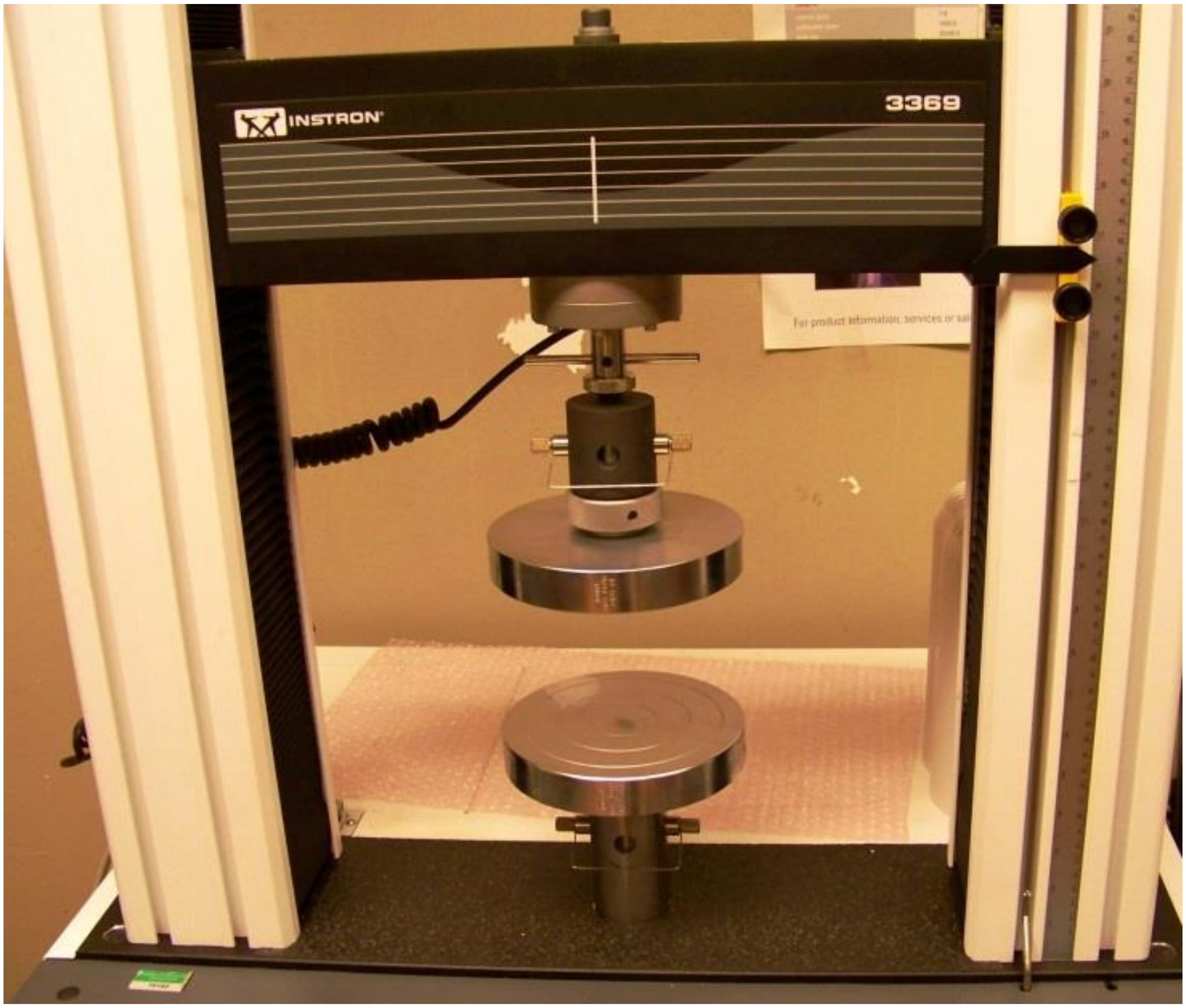

Figure S20

Instrument used for determination of bulk density of prepared polymers: helium pycnometry Micromeritics AccuPyc II 1340.

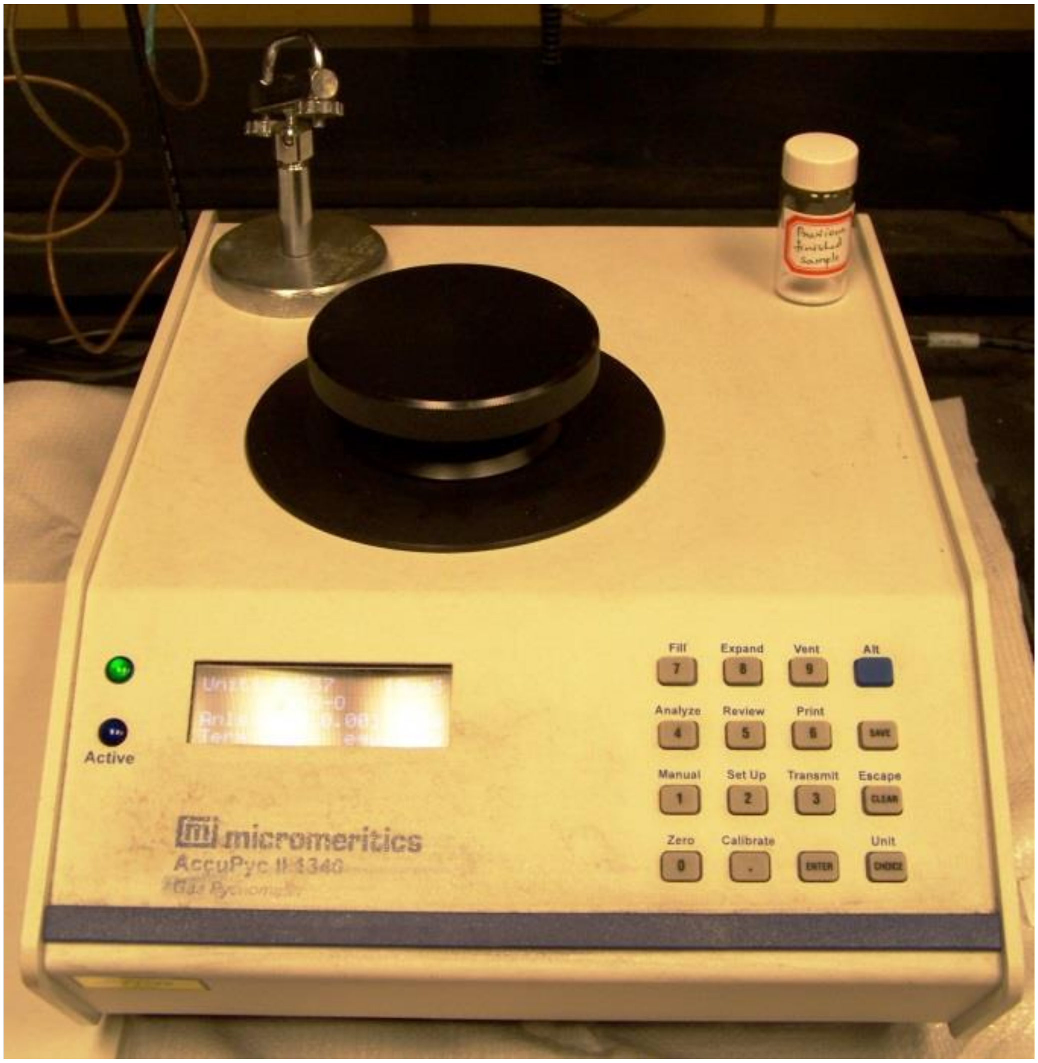

Figure S21

Micromeritics ASAP 2020 surface area and porosity analyzer used for attempt to measure the surface area and porosity of studied polymers.

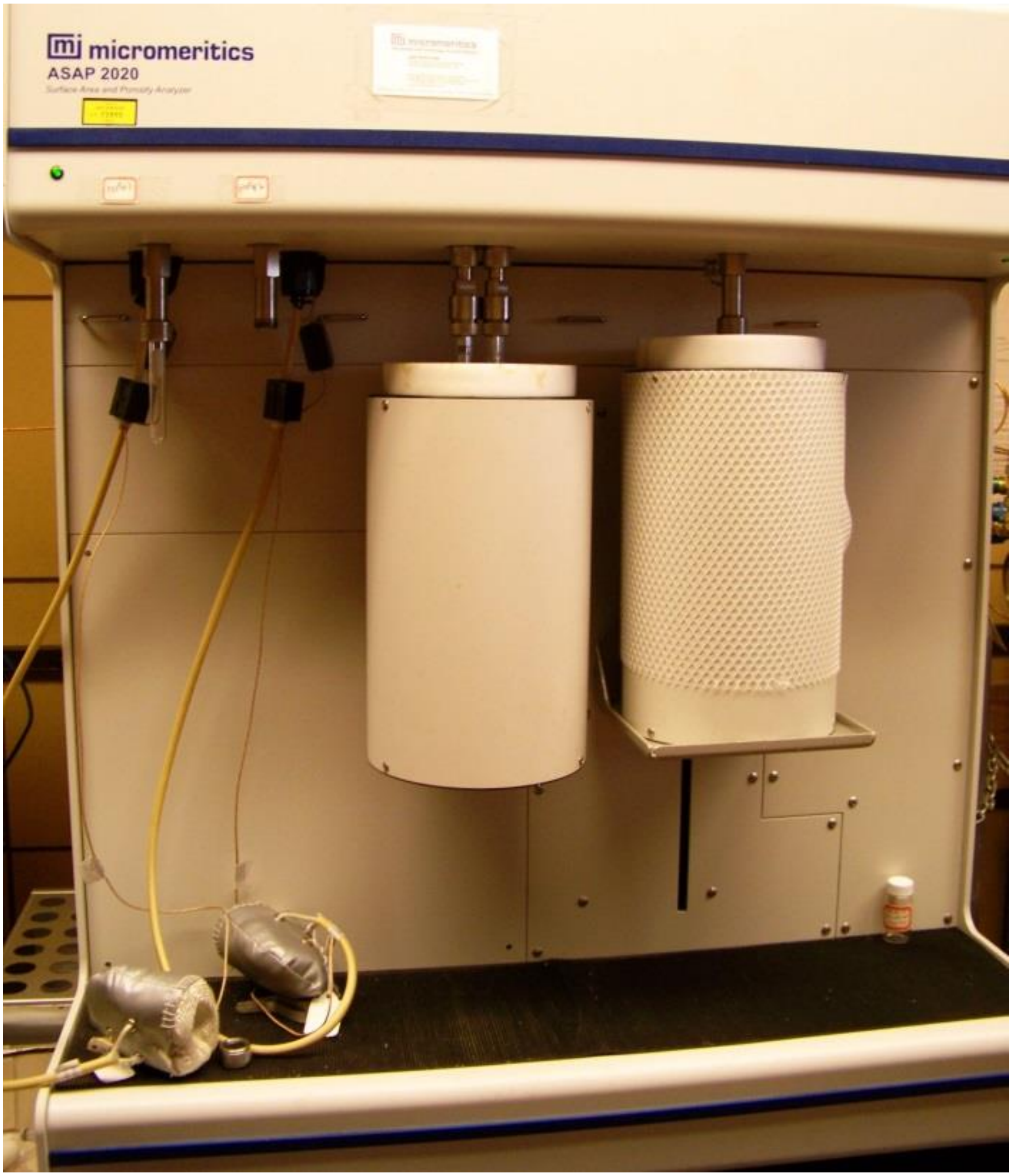

Figure S22

In vitro cytotoxicity studies results: photographs of HeLa (**A**) and WiDR (**B**) cells.

A1, B1 – initial cultures;

A2, B2 – after addition of 0.1 mM Ag(ACO) solutions;

A3, B3 – after treatment with 0.1 mM solution of control – cisplatin, $[Pt(NH_3)_2Cl_2]$.

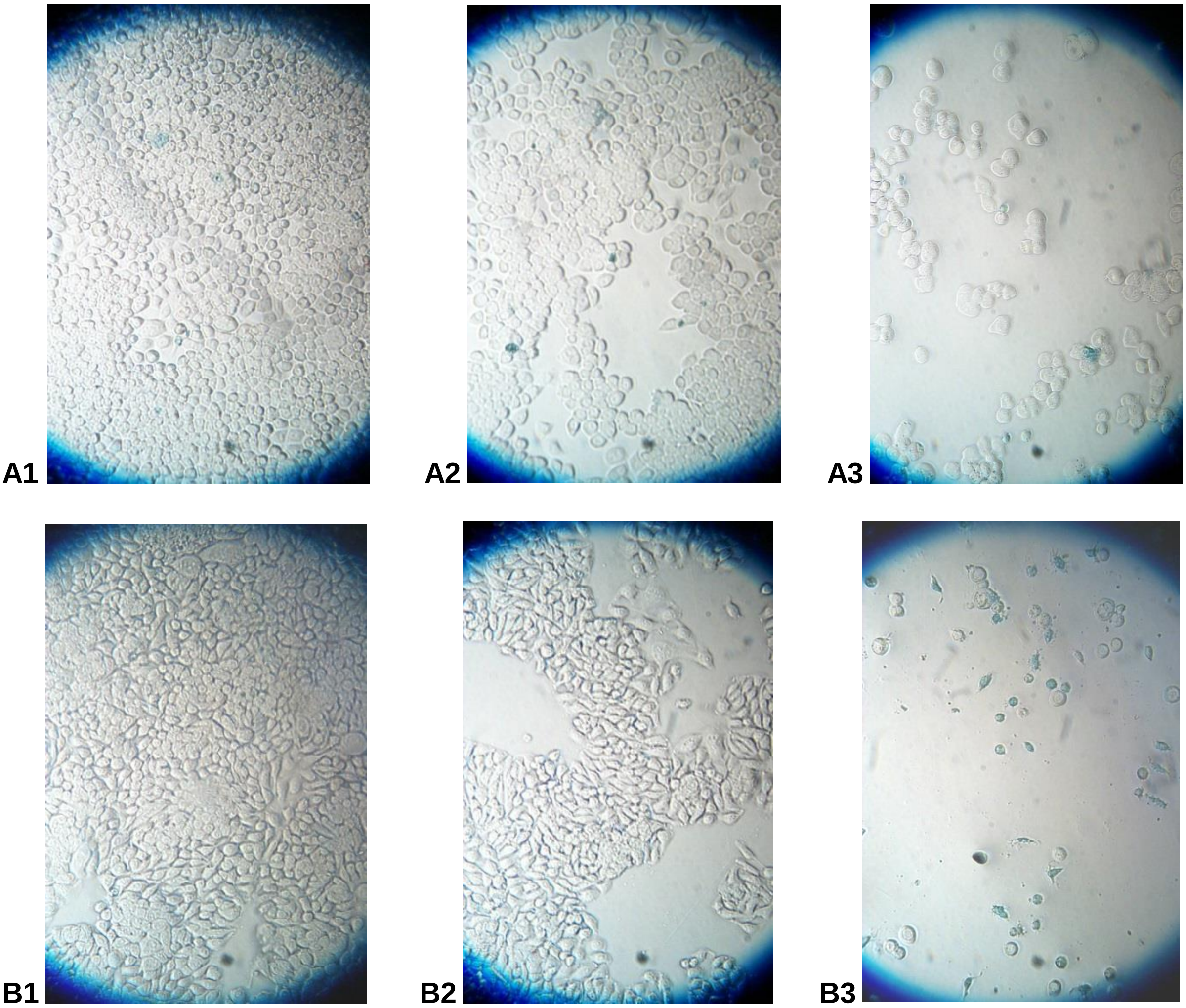

A1 **A2** **A3**

B1 **B2** **B3**
